# A Machine-Based Prediction Model of ADHD Using CPT Data

**DOI:** 10.3389/fnhum.2020.560021

**Published:** 2020-09-17

**Authors:** Ortal Slobodin, Inbal Yahav, Itai Berger

**Affiliations:** ^1^Department of Education, Ben-Gurion University, Beer-Sheva, Israel; ^2^Coller School of Management, Tel Aviv University, Tel Aviv, Israel; ^3^Pediatric Neurology, Assuta Ashdod University Hospital, Ashdod, Israel; ^4^Faculty of Health Sciences, Ben-Gurion University, Beer-Sheva, Israel

**Keywords:** attention-deficit/hyperactivity disorder, continuous performance test, machine learning, prediction, children

## Abstract

Despite the popularity of the continuous performance test (CPT) in the diagnosis of attention-deficit/hyperactivity disorder (ADHD), its specificity, sensitivity, and ecological validity are still debated. To address some of the known shortcomings of traditional analysis and interpretation of CPT data, the present study applied a machine learning-based model (ML) using CPT indices for the Prediction of ADHD.Using a retrospective factorial fitting, followed by a bootstrap technique, we trained, cross-validated, and tested learning models on CPT performance data of 458 children aged 6–12 years (213 children with ADHD and 245 typically developed children). We used the MOXO-CPT version that included visual and auditory stimuli distractors. Results showed that the ML proposed model performed better and had a higher accuracy than the benchmark approach that used clinical data only. Using the CPT total score (that included all four indices: Attention, Timeliness, Hyperactivity, and Impulsiveness), as well as four control variables [age, gender, day of the week (DoW), time of day (ToD)], provided the most salient information for discriminating children with ADHD from their typically developed peers. This model had an accuracy rate of 87%, a sensitivity rate of 89%, and a specificity rate of 84%. This performance was 34% higher than the best-achieved accuracy of the benchmark model. The ML detection model could classify children with ADHD with high accuracy based on CPT performance. ML model of ADHD holds the promise of enhancing, perhaps complementing, behavioral assessment and may be used as a supportive measure in the evaluation of ADHD.

## Introduction

Attention-deficit/hyperactivity disorder (ADHD) is one of the most common neurodevelopmental disorders (Barkley, 2015), with an estimated prevalence of 9.4% in USA children (Centers for Disease Control and Prevention, 2018). The rates of ADHD diagnoses have been rising in recent decades. In 2003, 7.8% of the USA children were diagnosed with ADHD, compared to 9.5% in 2007 and 11% in 2011–2012 (Danielson et al., 2018). ADHD is characterized by symptoms of inattention and/or impulsivity and hyperactivity, which can adversely impact the behavioral, emotional, and social aspects of life. In approximately 80% of children with ADHD, symptoms persist into adolescence and may continue into adulthood (Faraone et al., 2003).

Because early behavioral and developmental interventions for ADHD could improve outcomes (Sonuga-Barke and Halperin, 2010; Halperin et al., 2012), there is a need for reliable diagnostic ADHD markers that can be identified early in life.

ADHD diagnosis is based on the criteria of the Diagnostic and Statistical Manual of Mental Disorders (DSM), which are clinically judged and therefore are subjective to clinician and reporter’s bias (Rousseau et al., 2008; Berger, 2011). Diagnostic criteria bias may reflect socio-cultural influences on symptom manifestation and diagnostic procedures (for review see Slobodin and Masalha, 2020) as well as the substantial overlap between ADHD symptoms and other psychiatric, developmental and neurological conditions (e.g., learning disabilities, depression, anxiety; Nikolas et al., 2019). Thus, predicting ADHD impairment using objective, easy-to-collect variables by noninvasive methods might be useful as a supportive measure in the evaluation of ADHD and other neurological/psychiatric disorders (Na, 2019).

### Using CPT in the Diagnosis of ADHD

The continuous performance test (CPT) is one of the most popular objective measures of ADHD-related inattention and impulsivity (Edwards et al., 2007). CPTs usually include a serial presentation of visual or auditory target and non-target stimuli (numbers, letters, number/letter sequences, or geometric figures). Failing to respond to a target stimulus (“omission error”) is assumed to measure inattention. A response to a non-target stimulus (“commission error”) is considered to measure impulsivity. Other standard measures of CPT responses include the number of correct responses, the response time (RT), and the variability in RT.

Several studies have supported the utility of the CPT in the diagnostic process of ADHD (for review, Hall et al., 2016). For example, a recent study in the U.K. found that the QbTest (a computerized CPT combined with an infra-red camera to detect motor activity; Qbtech Limited) increased the speed and efficiency of ADHD clinical decision making without compromising diagnostic accuracy. Furthermore, the economic analysis revealed that the QbTest could increase patient throughput and reduce waiting times without significant increases in overall healthcare system costs (Hollis et al., 2018).

Despite its popularity, the utility of the CPT in the diagnostic process of ADHD has been long debated, due to its limited specificity, sensitivity, and ecological validity (Nigg et al., 2005; Toplak et al., 2013). Most of the methods used to discriminate between children with ADHD and typically developed children were based on standard statistical techniques, such as analysis of variance that were run on the data obtained from CPT measurements. These methods have led to inconsistent results between the researchers in the studies of ADHD children and adolescents (Hall et al., 2016). For example, an analysis of eight CPT studies revealed a wide variety in measures of sensitivity (9–88%) and specificity (23–100%) to ADHD (Pan et al., 2007). Similarly, a meta-analysis of 47 studies of CPT performance in children with ADHD found that the large effect sizes identified in previous research were significantly attenuated by unidentified true moderators or uncorrected artifacts, such as sampling error and measurement unreliability (Huang-Pollock et al., 2012). Traditional data analytic methods of CPT considerably limit the number of variables that can be used in a given analysis and, especially, the analysis of interactions. These methods of analysis also have limited ability to shed light on causality when the data are not based on randomized experimental designs (Deshpande et al., 2013). Most importantly, although standard approaches to CPT may distinguish clinical and non-clinical populations, they do not guarantee predictive optimality or parsimony in a data analysis-independent manner (Saxe et al., 2017). Taken together, the above findings emphasize the need to develop reliable validation techniques for both the clinical and research implications of CPT.

Machine learning (ML) is a rapidly emerging field that has allowed the exploitation of large datasets to generate predictive models. In “supervised learning,” machines develop ways of linking a target outcome from a set of predictors (“features”) in existing data. Such models may generalize to novel predictor data. In contrast to traditional statistical approaches, ML focuses on prediction rather than explanation (Hatton et al., 2019).

Availability and affordability of data collecting devices have opened doors for the use of ML to predict the likelihood of individuals developing a set of mental disorders such as depression, anxiety, autism, dementia, brain tumors, schizophrenia, psychosis, et cetera (Sen et al., 2018; Sakai and Yamada, 2019; Vieira et al., 2019). A growing number of supervised ML studies have been carried out on discriminating ADHD from control groups using the data obtained from electroencephalogram (EEG; Tenev et al., 2014), brain structural magnetic resonance imaging (MRI; Peng et al., 2013), MRI and functional magnetic resonance imaging (fMRI; Sen et al., 2018), Near-infrared spectroscopy (NIRS; Yasumura et al., 2017), and a combination of subjective and objective measures of ADHD (Emser et al., 2018). ML was also used to predict methylphenidate response in youth with ADHD using environmental, genetic, neuroimaging, and neuropsychological data (Kim et al., 2015). Although these models showed promising results in discriminating children and adults with ADHD from controls or other clinical conditions (e.g., autism spectrum disorders), their limited availability, high costs, and invasiveness hindered their widespread use.

### The Current Study

Behavioral diagnosis of ADHD is a time-consuming, multi-informant procedure that can be complicated by the overlaps in symptomatology. This complexity may lead to delayed diagnosis and treatment (Duda et al., 2016). Given the variation in causes and behavioral consequences of ADHD, there is no single test used to diagnose the disorder. Therefore, a diagnostic model of ADHD based on CPT performance holds the promise of enhancing, perhaps complementing, behavioral assessment. This study aimed to apply a ML-based model using a CPT for the prediction of ADHD.

## Materials and Methods

### Participants and Procedure

Participants were 458 children aged 6–12 years (mean = 8.68, SD = 1.77), 267 were boys (59%) and 191 girls (41%). Of them, 213 children were diagnosed with ADHD, and 245 were typically developed, children. No age differences were found between the ADHD and the non-ADHD groups (*M* = 8.62, *SD* = 1.83, and *M* = 8.72, SD = 1.71, respectively, *p* = 0.94). However, the rate of boys was significantly higher in the ADHD group than in the non-ADHD group (67% vs. 51%, respectively; $\chi_{\left(1,458\right)}^{2}$ = 10.84, *p* < 0.001).

Participants in the ADHD group were clinic-referred children recruited from out-patient pediatric clinics of a Neuro-Cognitive Centre, based in a tertiary care university hospital. Children were referred for ADHD evaluation by their pediatrician, general practitioner, teacher, mental health professional, or by their parents. All participants in the ADHD group met the criteria for ADHD, according to DSM-5 (American Psychiatric Association, 2013), as assessed by a certified pediatric neurologist. The diagnostic procedure included an interview with the patient and parents, medical/neurological examination as described by the American Academy of Pediatrics (AAP) clinical practice guidelines (Wolraich et al., 2011), and completing ADHD symptoms scales (DuPaul et al., 2016). All children were drug naïve.

Participants in the control group were randomly recruited from regular primary schools. Inclusion criteria for participants in the control group were: (1) the child scored below the clinical cut off point for ADHD symptoms on ADHD DSM Scales (American Psychiatric Association, 2013; DuPaul et al., 2016); and (2) an absence of academic or behavioral problems based on parents’ and teachers’ reports. Exclusion criteria for all participants were: intellectual disability, chronic use of medications, and primary psychiatric diagnosis (e.g., depression, anxiety, and psychosis).

All children were administered with the MOXO-CPT. The test was administered to children with ADHD during the process of clinical evaluation. In the non-ADHD group, the test was delivered by a member of the research team at the child’s school or home.

All participants agreed to participate in the study, and their parents provided written informed consent to the study, approved by the Helsinki Committee (IRB) of Hadassah-Hebrew University Medical Center Jerusalem, Israel. Participants were not compensated for their participation in the study.

### Measures

CPT performance—the current study used the MOXO-CPT^1^ version (Berger and Goldzweig, 2010). The MOXO-CPT (Neuro-Tech Solutions Limited) is a standardized computerized test designed to diagnose ADHD-related symptoms. The MOXO-CPT task requires the child to sustain attention over a continuous stream of stimuli and to respond to a prespecified target. However, in contrast to other existing CPTs, the test includes visual and auditory stimuli serving as measurable distractors. The test’s validity and utility in distinguishing children and adolescents with ADHD from their typically developing peers were demonstrated in previous studies (Berger et al., 2017; Slobodin et al., 2018).

**Figure 1:**
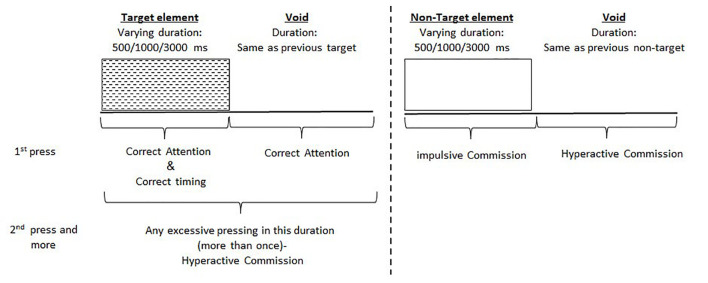
Definition of the timeline. Target and non-target stimuli were presented for 500, 1,000 or 3,000 ms. Each stimulus was followed by a void period of the same duration. The stimulus remained on the screen for the full duration regardless of the response. Distracting stimuli were not synchronized with target/non-target’s onset and could be generated during target/non-target stimulus or the void period.

The test consisted of eight stages (levels). Each level consisted of 53 trials (33 target and 20 non-target stimuli) and lasted 114.15 s. The total duration of the test was 15.2 min. In each trial, a stimulus (target or non-target) was presented in the middle of the computer screen for durations of 0.5, 1, or 3 s and was followed by a “void” of the same duration (Figure 1). This method enabled us to distinguish accurate responses performed in “good timing” (quick and correct responses to the target performed during stimulus presentation) from accurate but slow responses (correct responses to the target performed after the stimulus presentation; during the void period). These two aspects of timing correspond to the two different deficiencies typical to ADHD; responding quickly and responding accurately (National Institute of Mental Health, 2012). The child was instructed to respond to the target stimulus as quickly as possible by pressing the space bar once and only once. The child was also instructed not to respond to any other stimuli but the target, and not to press any other key but the space bar.

Both target and non-target stimuli were cartoon pictures free of letters or numbers. Also, the test included six different environmental distractors, each of them could appear as pure visual (e.g., three birds moving their wings), pure auditory (e.g., birds singing), or as a combination of visual and auditory stimuli (birds moving their wings and singing simultaneously). Each distractor was presented on the screen for a different duration ranging from 3.5 to 14.8 s, with a constant interval of 0.5 s between two distractors.

For each child, four CPT indices were recorded: attention (number of correct responses to target stimuli, including the rate of omission errors), Timeliness (correct responses to target stimuli conducted on accurate timing), Hyperactivity (a measure of motor activity) and Impulsiveness (responses to non-target stimuli, including the rate of commission errors). Test administration time, namely, the day of the week (DoW) and time of day (ToD), was also recorded. A full description of the MOXO-CPT test is provided in Supplementary Material (Appendix 1).

### Data Analysis

Data analysis included four stages: (a) data exploration and processing; (b) data partition and manipulation of training; (c) model fitting; and (d) ADHD prediction and evaluation.

**Figure 2:**
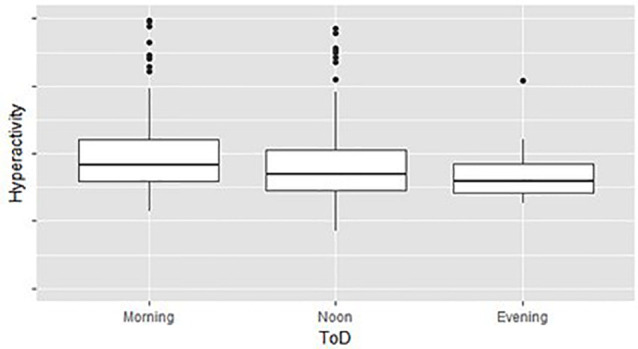
Hyperactivity as a function of time of day (ToD) in children without attention-deficit/hyperactivity disorder (ADHD) group differences are significant (*F* = 8.26, *p* < 0.01).

(1) Data exploration and processing—following the descriptive data analysis outlined in the method section, two sources of inherent biases were evident, which we denoted: across the level and within level imbalances.
  Across level imbalances—demographic and test time variables were imbalanced. For example, the total number of children that performed the MOXO-CPT during Mondays was significantly higher than the number of children that performed the test during any other DoW. The lowest number of tests was conducted on Saturdays. Imbalanced designs concerning the number of observations per level were shown to be highly susceptible to statistical biases, such as heteroscedasticity (Milliken and Johnson, 2009). Moreover, our data showed significant differences in MOXO-CPT performance across various DoW, ToD, and child’s ages, suggesting that across level imbalances may be associated with observed between or within-group differences in MOXO-CPT performance. Figure 2 illustrates different hyperactivity levels in children without ADHD as a function of the time of the day they performed MOXO-CPT. As seen in Figure 2, the level of hyperactivity significantly decreased with the time of the day (*F* = 8.26, *p* < 0.01).
  Within level imbalance—diagnostic class within demographic and test time levels were also shown to be imbalanced. For example, the ADHD group significantly differed from the non-ADHD group in their gender distribution, with more boys in the ADHD group than in the non-ADHD group ($\chi_{\left(1,458\right)}^{2}$ = 10.84, *p* < 0.001). Moreover, for some factor levels, such as DoW and ToD, the data included only children from one diagnostic group, but not the other; administration of the MOXO-CPT during the weekend was evident only among the non-ADHD group. Also, all children in the ADHD group (with one exception) performed the MOXO-CPT during the morning hours.
  Within-level imbalance results in a statistical bias that is closely related to the renowned self-selection bias (Heckman, 1990), a biased caused by participants choosing themselves into treatment groups rather than assigned randomly. In the context of the current study, the time of MOXO-CPT administration was not randomized, and group affiliation was not matched, leading to differences in gender, age, DoW, and ToD distribution between groups. This imbalance may impose a significant prediction bias if demographic and test time controls were not matched before deploying an ML model on the CPT data.
  To quantify the statistical biases in our data, we conducted a retrospective factorial fitting (Loy et al., 2002). This technique sorts observations into groups according to their factor levels—a total of 7 [age] × 2 [gender] × 7 [DoW] × 3 [ToD] = 194 groups. Following the procedure proposed by Yahav et al. (2016), we then merged groups in which the impact of ADHD on test performance was statistically equal, thus reducing the number of groups to 28 groups. We then computed the number of records in each group: *n_i_* (across-level imbalance), and the fraction of children with ADHD *p_i_(ADHD)* (within-level imbalance). Groups in which the fraction of children with ADHD equaled to either 0 or 1, were removed from the dataset, as the impact ADHD on test performance within these groups was unquantifiable. The processed data contained 445 observations (97% of the unprocessed data).
(2) Data partition and manipulation of training—as customary in predictive analysis (Shmueli, 2010), we partitioned the data randomly to training and holdout. The training set consisted of 60% of the records and was used for model training. The holdout set held the rest of the records (40%) and was used for model evaluation.
  We manipulated the training set to correct the inherent biases. Specifically, we bootstrapped the inflated number of training records (*Ñ* = 5,000) using bootstrap sampling with repetition, following the principles of the Synthetic Minority Oversampling Technique (SMOTE). SMOTE algorithm is a kind of random oversampling algorithm which generates new synthetic samples by analyzing neighbors of minority samples (Chawla et al., 2002; He and Garcia, 2009). We set the oversampling probability per record as inverse to its appearance in the data concerning levels (1 − *n*_i/N_) and diagnosis [1 − *p*_i_*(ADHD)*] for children with ADHD, and *p_i_(ADHD)* for children without ADHD. This sampling procedure generated a training data set that was: (1) synthetically large enough to allow the use of robust ML techniques that operate on large amounts of data; and (2) balanced within- and across- levels, thus was free of statistical biases. The holdout set remained untouched, to allow a fair evaluation of the prediction model.
(3) Model fitting—in this stage, we trained an ML prediction model *f* to the training set that mapped all or a subset of the test performance measures (Attention, Timeliness, Hyperactivity, and Impulsiveness) and additional control variables (age, gender, DoW, and ToD) to the diagnosis class:

(1) p(diagnosis=ADHD)=f(test performance, controls)

Specifically, *f* in our analysis is either a random forest (Hothorn et al., 2006) or Neural Network with cross-validation on 100 folds^2^.

**Figure 3:**
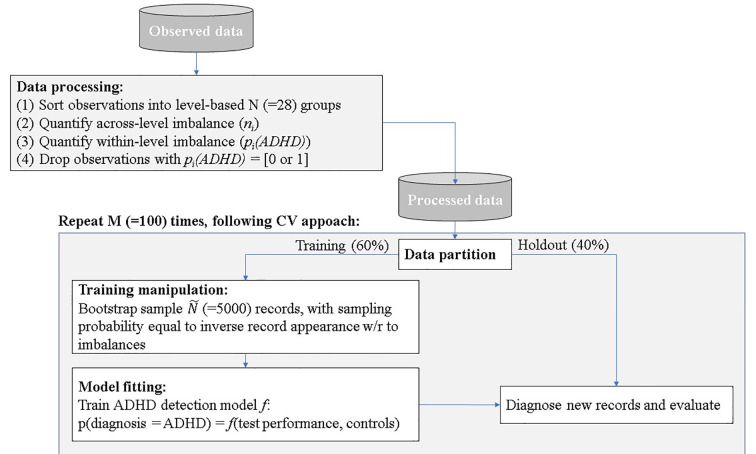
Proposed detection procedure.

(4) ADHD prediction and evaluation—in this stage, we used the training model to predict the detection of ADHD in holdout records. That is, we computed the accuracy, sensitivity, and specificity of our ML model comparing to clinical diagnosis. We repeated steps 3 and 4 (data partition and training manipulation, model fitting, and ADHD detection and evaluation) 100 times, generating a different training-validation random split as each repetition, to compute conference intervals for the accuracy measures. The detection procedure is summarized in Figure 3.

## Results

We examined different subsets of MOXO-CPT performance indices (Attention, Timeliness, Hyperactivity, and Impulsiveness) and control variables (age, gender, Dow and ToD) as ADHD predictors in children who were diagnosed with ADHD, using the “gold standard” clinical criteria. The gold standard of ADHD diagnosis was based on the DSM-V criteria for ADHD (American Psychiatric Association, 2013) and the AAP clinical practice guideline (Wolraich et al., 2011).

As a benchmark, we trained function *f* in Equation (1) on a training set derived from the unprocessed (original) data. Since the unprocessed data contained inherent statistical biases caused by level-imbalance (within and across), it was impossible to use the control variables as reliable predictors in the benchmark model.

The results of the random forest and the Neural Network (NN) techniques are presented in Table 1; *t*-tests analyses for paired samples revealed that the differences between the benchmark model and the proposed models (in both random forest and NN techniques) were significant (*p* < 0.05). However, no significant differences were found between the results of the random forest technique and the NN technique (*p* > 0.05). As seen in the table, NN provides, on average, higher rates of accuracy and sensitivity to ADHD, compared to the random forest technique. However, the confidence intervals in the NN model are higher, indicating non-robust cases and higher risk prediction errors. Given the increased robustness of the random forest technique and the fact that it was not significantly different from NN in all observed measures (overall accuracy, sensitivity, and specificity), it was chosen as the preferred method.

**Table 1:** Accuracy, sensitivity and specificity of machine learning (ML) in attention-deficit/hyperactivity disorder (ADHD) classification.

	Model’s name	Diagnosis predictors	Mean performance (SD)^c^					
			Overall accuracy (%)	95% CI	Sensitivity%	95% CI	Specificity (%)	95% CI
Benchmark	MOXO	MOXO-CPT scores:	65	59%, 71%	65	39%, 91%	65	43%, 87%
Proposed model *f* = Random forest	MOXO	MOXO-CPT scores^a^	81	77%, 85%	84	78%, 90%	79	71%, 87%
	MOXO w/Controls	MOXO-CPT scores considering control variables^b^	87	81%, 93%	89	83%, 95%	84	76%, 92%
Proposed model *f* = Neural network	MOXO w/Controls	MOXO-CPT scores considering control variables^b^	89	79%, 99%	95	73%, 100%	76	26%, 100%

*^a^In this prediction model, all four MOXO-CPT indices (z-scores) were used as independent variables. ^b^In this prediction model, all four MOXO-CPT indices (z-scores) and four control variables (age, gender, time of day, day of week) were used as independent variables. ^c^The differences between the benchmark model and the proposed models (random forest method and NN) were all significant (*p* < 0.05). No significant differences were found between the results of random forest technique and the NN technique (*p* > 0.05)*.

As shown in the table, the best performance of the random forest technique (over 87% accuracy, 89% sensitivity and 84% specificity) was achieved by applying the proposed method to predict ADHD, using the four CPT performance indices and four controls as predictors. This performance was 34% higher than the best-achieved accuracy of the benchmark^3^. Prediction solely based on MOXO-CPT performance, without the use of additional controls, had an accuracy of 81% (84% sensitivity and 79% specificity), an improvement of 24.6% compared to the benchmark. Among the four CPT performance indices, Impulsiveness under the benchmark model had the highest ability to rule out ADHD in children (Specificity = 89%, significantly higher than all other models).

As an alternative to the SMOTE rebalancing technique, we also examined the impact of the under-sampling technique to balance between the control and the ADHD groups. This rebalancing technique revealed an overall accuracy of 73% (%95 CI = 63 to 83%), a sensitivity rate of 79% (%95 CI = 57 to 100%), and a specificity rate of 66% (%95 CI = 42 to 90%). As seen, there are considerable differences between the results of the two rebalancing techniques. While both techniques addressed the four potentially confounding variables (DoW, time of the day, gender, and age) in the same manner (same size, unprocessed), they differ in their ability to capture information about the majority class. Specifically, the disadvantage of the under-sampling technique is that removing modules may cause the training data to lose important information related to the majority class. The SMOTE technique was proposed to combat this disadvantage by creating artificial data based on the feature space (rather than the data space) similarities from the minority modules (Tantithamthavorn et al., 2018). The advantage of the SMOTE is that it leads to no information loss and ensures that even small-size confounding effects are not overlooked.

A comparison of the model’s performance as a function of data size is presented in Supplementary Material (Appendix 2). The models are all trained on a balanced training set and predicted into a non-balanced validation set. Notably, the increase in the training size was synthetic and resulted from repeating the same records several times, following the bootstrap technique. The validation score remained constant (40%).

Table 2 presents the random forest feature importance. In the current study, we used the accuracy-based importance as a measure of variable importance in the random forest. In this measure, each tree has its out-of-bag sample that is used to calculate the importance of a specific variable. In the first step, the prediction accuracy of the out-of-bag sample is measured. Then, the values of the variable in the out-of-bag-sample are randomly shuffled, as all other variables are kept unchanged. Finally, the decrease in prediction accuracy on the shuffled data is measured. The mean decrease in accuracy across all trees is reported. Variables with high importance have a significant impact on the outcome values (Breiman, 2001, 2002). Notably, the displayed values indicate the relative importance of each feature when making a prediction and not absolute importance.

**Table 2:** Random forest feature importance.

Predicting variables	Random forest feature importance
*Z*-score timeliness	0.170
*Z*-score hyperactivity	0.049
*Z*-score attention	0.041
*Z*-score impulsiveness	0.041
Gender	0.037
Day of the week	0.039
Child’s age	0.028
Time of the day	0.004

*These results are not identical to variable coefficient, and the interaction between the variables could not be observed in the RF model*.

## Discussion

The current study applied a machine learning-based predictive model using CPT indices for the prediction of ADHD in children aged 6–12 years.

Our findings demonstrated that the ML proposed model performed better and had a higher accuracy than the benchmark approach that used clinical data only. We also found that the CPT total score (including all four indices: Attention, Timeliness, Hyperactivity, and Impulsiveness), and all four control variables (age, gender, DoW, and ToD), provided the most salient information for discriminating between children with ADHD and their typically developed peers. This model had an accuracy rate of 87%, a sensitivity rate of 89%, and a specificity rate of 84%. Using this model increased the performance by 34% compared to the benchmark approach.

The improvement in the model’s prediction accuracy after quantifying for cross-level and within-level imbalances suggests that such statistical biases may affect the discriminative validity of the CPT. These findings are in line with previous research that pointed to the influence of age (Berger et al., 2013; Slobodin et al., 2018) and gender (for review, Hasson and Fine, 2012) on CPT performance. Although studies focusing on the effect of time administration on CPT performance are currently scarce, there is evidence to support the importance of accounting for inter-day and intraday variations when comparing CPT performance of children with ADHD to that of their typically developed peers (van der Heijden et al., 2010). For example, Imeraj et al. (2012) showed that children with ADHD (with or without a co-morbid oppositional defiant disorder) significantly differed from healthy controls in their cortisol profiles across the day. Such variations may underline group differences in arousal mechanisms and may also affect CPT performance (Wang et al., 2017).

Comparing our results to other ML methods previously used to discriminate between ADHD and controls suggests that an ML model based on CPT data holds the promise of discriminating children with ADHD from controls, even when compared to more invasive or expensive approaches. For example, Yasumura et al. (2017), who used near-infrared spectroscopy to quantify the change in prefrontal cortex oxygenated hemoglobin during reversed Stroop task, found an overall discrimination rate of 86.25%, with a sensitivity of 88.71% and a specificity of 83.78%. Likewise, using MRI data, Peng et al. (2013) found an ADHD prediction accuracy of 90.18%. Recently, Emser et al. (2018) developed an ML prediction model of ADHD based on subjective and objective measures of ADHD, including a CPT (Quantified Behavior Test for adolescents and adults). Their results showed that the objective measures had an overall 78% accuracy and that the combined model accuracy of the objective and subjective measures was 86.7%.

Using ML CPT-based model to predict ADHD offers several clinical and practical advantages. First, this model provides an easy-to-administer, affordable, non-invasiveness measure of ADHD-related symptoms. Second, it has an extremely fast discrimination speed and satisfactory high classification accuracy. In particular, the observed high sensitivity rates (89%) may improve clinicians’ ability and confidence in ruling out ADHD. Excluding ADHD when it is not present is very important given the complicated, time-consuming, and expensive process of ADHD diagnosis (Hall et al., 2017). Third, ML models can handle various demographic and procedural variables, that may hinder CPT’s discriminative utility, to make an objective prediction (Emser et al., 2018). Finally, the current ML model is based on CPT performance that is obtained under the presence of environmental distractors, thus enables the assessment of the child’s cognitive performance in an ecologically-valid environment (Barkley, 2015).

While this study does not support the viability of solely CPT-based algorithms for establishing a diagnosis of ADHD, it presents a step towards the goal of precision medicine in psychiatry. The ML model proposed here may inform the development of a decision-support module that utilizes the best-performing model, thus improving the quality of care for ADHD (Carroll et al., 2013).

Several limitations of this study should also be considered. The current study only examined standard CPT variables, including inattention, RT, hyperactivity, and impulsivity. However, ADHD is associated with additional cognitive deficits that may affect CPT performance, such as distractibility and fatigue over time (Pelham et al., 2011; Bioulac et al., 2012). Future investigation should use these additional variables with the current classification method to further improve ADHD classification performance. Also, our sample was limited to clinically-referred children with a definite ADHD diagnosis while excluding children with suspected ADHD (whose symptoms may not reach the threshold for diagnosis). Given that the time gap between initial suspicion and diagnosis could reach a year or even more, the ability of the ML model to provide preliminary risk evaluation and/or pre-clinical screening is of considerable significance in terms of timely intervention (Duda et al., 2016). Another limitation of the study is related to its reliance on a single recruitment center for children with ADHD. Although the current sample was derived from a tertiary care university hospital that provides services to the general population, this fact limits our ability to generalize our results to different populations. Also, there is limited information about participants’ demographic, cognitive, and clinical characteristics, such as IQ level and ADHD subtypes, which can be associated with CPT performance (Mahone et al., 2002; Collings, 2003). Given our limited information about participants’ cognitive and personal characteristics, there was no way to rule out the possibility that some participants were not motivated to optimize their CPT performance.

Our model is also limited by the relatively small number of candidate classification features that were tested in the adaptive models (four CPT indices, two demographic variables, and two condition variables). Future ML research in ADHD prediction should expand this examination to include a broader range of clinical, behavioral, and demographic variables, including education, ethnicity, socio-economic status, psychiatric-co-morbidities, and medication use. Finally, while the predictive accuracy of ML models can be satisfactory, a naive implementation of ML without careful validation may have adverse consequences. An ML algorithm may replicate past decisions, including biases around ethnicity and gender, that may have affected the clinical judgment. Therefore, model extrapolation should be avoided until such biases are corrected (Saria et al., 2018).

## Conclusions

Previous studies using standard, traditional analyses of CPT data provided evidence for the ability of the test to differentiate between children with ADHD and their typically developing peers. Nevertheless, these approaches were limited in their ability to optimally predict ADHD, to draw a causal inference, and to include multiple variables in a given analysis (Saxe et al., 2017). Thus, developing reliable validation techniques for both clinical and research implications of CPT is of high importance.

Our result showed that ML diagnostic model could predict ADHD in children with high accuracy based on CPT performance indices. This model performed better than any achieved benchmark model to CPT. Using an ML model based on CPT may provide good classification accuracy for supporting ADHD diagnoses in children and encourages the use of the CPT as a quick, cost-effective, and accurate decision-making tool in the ADHD diagnosis process (Hollis et al., 2018).

## Data Availability Statement

The raw data supporting the conclusions of this article will be made available by the authors, without undue reservation.

## Ethics Statement

The studies involving human participants were reviewed and approved by the Helsinki Committee (IRB) of Hadassah Hebrew University Medical Center, Jerusalem, Israel. Written informed consent to participate in this study was provided by the participants’ legal guardian/next of kin.

## Author Contributions

IB recruited the patients and performed clinical evaluations. IY performed data analyses. OS, IY, and IB contributed equally to the development of study design, integration of findings, and writing the manuscript.

## Conflict of Interest

OS and IB have previously served on the scientific advisory board of NeuroTech Solutions Limited.

The remaining author declares that the research was conducted in the absence of any commercial or financial relationships that could be construed as a potential conflict of interest.

## Footnotes

^1^The term “MOXO” derives from the world of Japanese martial arts and means a “moment of lucidity.” It refers to the moments preceding the fight, when the warrior clears his mind from distracting, unwanted thoughts, and feelings.

^2^We have also conducted analyses with additional ML models, including kNN, Logistic regression and Adaptive boosting. The best performance was achieved by Neural Network (overall accuracy of 0.89, compared to 0.68 of the benchmark model), and the lowest by logistic regression (0.83, compared to 0.61).

^3^The 34% improvement was calculated with respect to 65% performance level of the benchmark.
